# Association between blood lead, cadmium, selenium levels and hyperlipidemia: A population-based study

**DOI:** 10.1371/journal.pone.0306573

**Published:** 2024-08-15

**Authors:** Yangping Zhuang, Yu Wang, Peifen Sun, Jun Ke, Feng Chen

**Affiliations:** 1 Shengli Clinical Medical College of Fujian Medical University, Fujian Medical University, Fuzhou, China; 2 Department of Emergency, Fujian Provincial Hospital, Fuzhou, China; 3 Fujian Provincial Key Laboratory of Emergency Medicine, Fuzhou, China; Hamadan University of Medical Sciences, School of Public Health, ISLAMIC REPUBLIC OF IRAN

## Abstract

**Background:**

There are limited epidemiological investigations of blood metal levels related to hyperlipidemia, and results indicating the association between blood lead (Pb), cadmium (Cd) and selenium (Se), and lipid biomarkers have been conflicting.

**Methods:**

We included populations for which NHANES collected complete data. Multivariate logistic regression and subgroup analyses were conducted to ascertain the relationship between blood Pb, Cd, and Se levels and hyperlipidemia. Nonlinear relationships were characterized by smoothed curve fitting and threshold effect analysis.

**Results:**

5429 participants in all, with a mean age of 53.70 ± 16.63 years, were included; 47.1% of the subjects were male, and 3683 (67.8%) of them had hyperlipidemia. After modifying for variables with confounders in a multivariate logistic regression model, we discovered a positive correlation between blood Pb and Se levels and hyperlipidemia (Pb: OR:2.12, 95% CI:1.56–2.88; Se: OR:1.84, 95% CI:1.38–2.45). Gender, age, smoking status, alcohol use status, hypertension, diabetes, and body mass index were not significantly linked with this positive correlation, according to subgroup analysis and interaction test (***P*** for interaction>0.05). Positive correlations between blood Pb, Cd, and Se levels and the risk of hyperlipidemia have been found using smooth curve fitting.

**Conclusions:**

This study demonstrates that higher blood levels of Pb, Cd, and selenium are linked to an increased risk of hyperlipidemia.

## Introduction

Hyperlipidemia is a metabolic disorder defined by elevated levels of lipids in the blood. Variations in triglyceride, total cholesterol, low-density lipoprotein, and high-density lipoprotein cholesterol levels often indicate hyperlipidemia [1]. Throughout both sides of Europe and America, the prevalence of hyperlipidemia, one of the main risk factors for cardiovascular and metabolic disorders, is increasing. The subject matter includes, but is not restricted to, several illnesses associated with hyperlipidemia [2]. Data from the NHANES study show that roughly 50% of Americans struggle with hyperlipidemia [3]. Throughout the world, dyslipidemia is one of the prerequisites for circulatory metabolic diseases, and hyperlipidemia increases the risk of cardiovascular disease, which can cause stroke as well as death [4–6].

Dyslipidemia can be caused by various circumstances, such as inherited conditions, western dietary regimens, environmental influences, and more [7]. The amount of metals in our surroundings has constantly increased over time because of the rapid growth of the mining, processing, aluminum smelting, and refining industries [8, 9]. Individuals consume and absorb elements daily through various routes, such as contact with their bodies with products made from chemicals, including paints, ceramics, and plumbing materials, assimilation into the food chain, and inhaling water and airborne particles [10, 11]. Because metals are difficult to break down, they are absorbed into the circulatory system and progressively accumulate there [12–14]. Further, it is a form of metal contamination now well-acknowledged as an environmental risk [15–17]. Studies on the Korean population indicate that exposure to metallic substances increases the incidence of coronary artery bypass graft disease among Koreans [18].

Extremely rare to find epidemiologic studies that connect exposure to heavy metal levels to hyperlipidemia, and the evidence supporting this relationship is ambiguous. For example, exposing obese rats to metals has been verified in an anterior animal experiment to cause oxidative stress, changes in lipid metabolism, and, eventually, dysfunction of the processes that regulate lipids [19]. A survey of the general population in Nigeria found that exposure to lead-based paint was associated with lipid changes and an increased risk of developing cardiovascular disease [20]. Those exposed to metal-based lead were also far more likely than regulates to demonstrate high levels of low-density lipoprotein but not high levels of cholesterol or triglycerides in high-density lipoprotein [21]. Outcomes from animal experiments showed that although the administration of cadmium exacerbated severe hyperlipidemia and fatty liver changes in zebrafish, selenium deficit boosted the transcription of Apo B [22]. Application of selenium at a concentration of one milligram per millimeter resulted in the downregulation of Apo B transcription [23]. As a result, more research is required to determine the association between blood metal levels following exposure to heavy metals and hyperlipidemia. Measuring blood levels of lead, cadmium, and selenium metals after exposure to heavy metals, one of the most reputable indicators of hyperlipidemia management in the clinic may help clarify the direction for further fundamental research.

Based on data from four cycles of the National Health and Nutrition Examination Survey, which ran from 2011 to 2018, this study aimed to investigate the relationship between blood lead, cadmium, and selenium metal levels and hyperlipidemia. The goal was to look for information that might clarify the ambiguous relationships between lipid biomarkers and metal lead, cadmium, and selenium exposures discovered in previous studies.

## Materials and methods

### Study design and population

The National Center for Health Statistics oversees NHANES, a complicated stratified multistage population-based cross-sectional survey. Its goal is to evaluate the dietary and physical health of children and adults in the US. Every two years, data for the NHANES program are gathered from a nationally representative sample utilizing home interviews and physical exams. Visit http://www.cdc.gov/nchs/nhanes/index.htm for additional details on the NHANES Continuous Survey design. The National Center for Health Statistics Ethics Review Board blessed all survey methodologies, and all participants signed informed consent before any data was collected.

**Fig 1:**
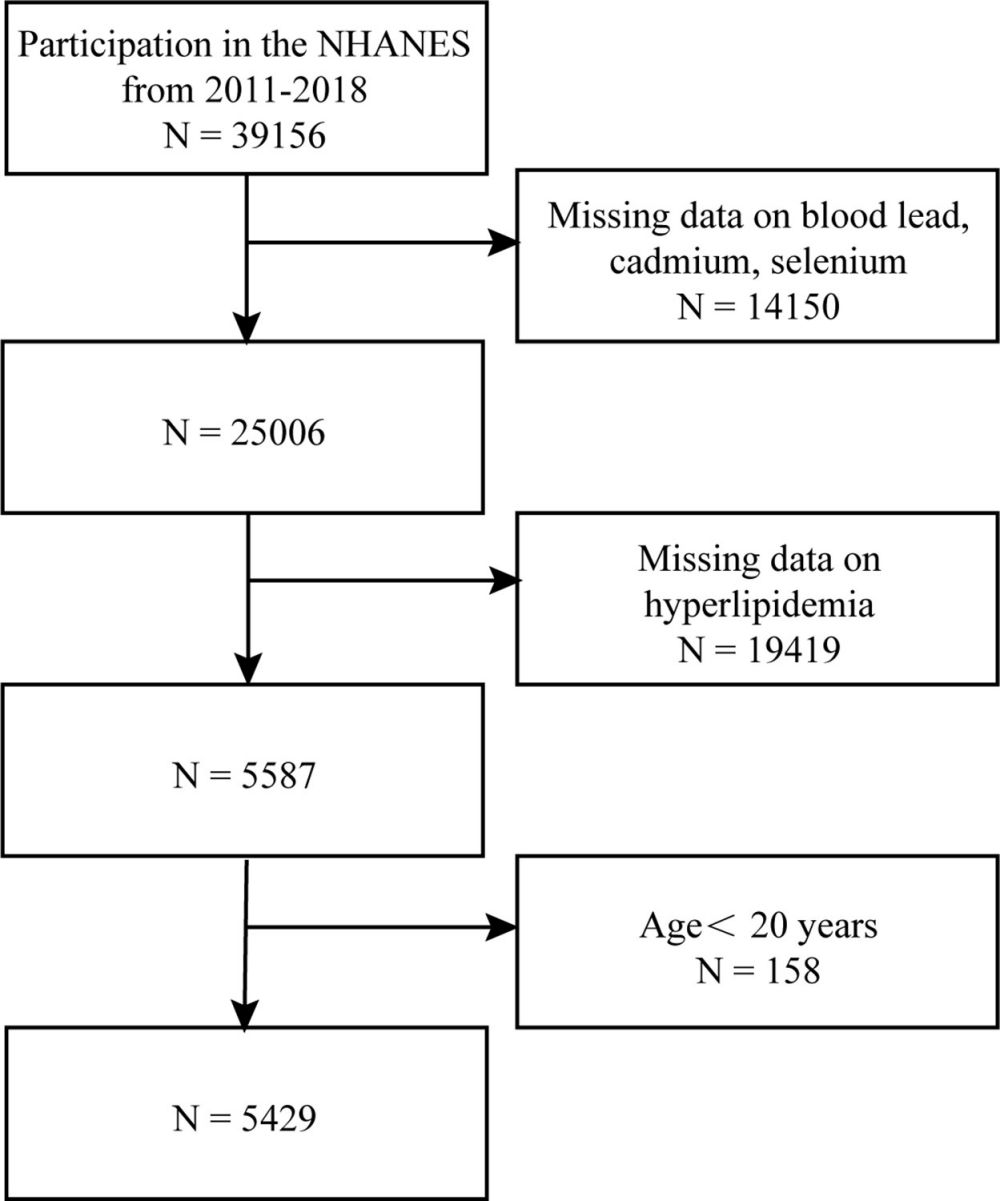
Flowchart for study population selection.

We integrated and examined demographic, hyperlipidemia, and blood metals data from four NHANES cycles (2011–2012, 2013–2014, 2015–2016, and 2017–2018). Between 2011 to 2018, 39,156 individuals in total were registered for the NHANES. We eliminated participants whose metal levels were missing, whose hyperlipidemia data were lacking, and those under 20 years old. Finally, this study comprised 5429 individuals, all 20 years or older (Fig 1).

### Assessment of hyperlipidemia

Hyperlipidemia is defined as total cholesterol 200 mg/dL, triglycerides 150 mg/dL, HDL (40 mg/dL for males and 50 mg/dL for women), or low-density lipoprotein 130 mg/dL according to the National Cholesterol Education Program (NCEP) Adult Treatment Panel III (ATP 3) [24]. Furthermore, those who disclosed utilizing cholesterol-lowering drugs were categorized as hyperlipidemia.

### Blood trace elements test

The technique for determining blood Pb, Cd, and Se levels in blood advances over earlier techniques. The National Center for Environmental Health and the Centers for Disease Control and Prevention received whole blood samples drawn, processed, stored, and transported for examination. The samples are diluted in the lab before being kept at -20°C. Inductively coupled plasma dynamic reaction cell mass spectrometry (ICP-DRC-MS) was used to determine the levels of lead, cadmium, and selenium in the blood. Visit https://wwwn.cdc.gov/Nchs/Nhanes/2017-2018/PBCD_J.htm for a thorough explanation of the NHANES laboratory procedures. Like the Westgard Rules, these blood trace element findings fulfill the Laboratory Sciences Division’s Quality Control and Quality Assurance performance criteria for accuracy and precision. Under the limit of detection (LOD), blood levels of Pb, Cd, and Se were substituted by the LOD divided by the square root of two. Blood Pb, Cd, and Se levels were all log-ln converted to resemble a normal distribution.

### Covariates

Using a computer-assisted personal interviewing (CAPI) system, trained interviewers collected demographic data from household interviews and mobile examination center (MEC) interviews. The covariates in this study include age, gender, race(Mexican American, Other Hispanic, Non-Hispanic White, Non-Hispanic Black, Other Races), marital status(Married/Living with partner, Widowed/Divorced/Separated, Never married), education level(Less than 9th grade, 9-11th grade, High school graduate/GED or equivalent, Some college or AA degree, College graduate or above), smoking status, alcohol use status, body mass index, hypertension, diabetes, family income and family size(total number of people in the family). At least four or five drinks are considered to be consumed daily. Twelve ounces of beer, 5 ounces of wine, or 1.5 ounces of liquor make up one drink. A lifetime of at least 100 cigarettes is required to be considered a smoker. Weight divided by height squared was used to compute body mass index (BMI), which was then classified as not overweight/obesity (BMI<25 kg/m^2^), overweight (25 kg/m^2^<BMI<30 kg/m^2^), and obese (BMI>30 kg/m^2^). Participants’ self-reported medical diagnoses allowed us to learn about their past experiences with hypertension and diabetes.

### Statistical analysis

A complex multi-stage sampling process was used to survey the NHANES database using suitable sampling weights and complicated multi-stage cluster surveys. The Centers for Disease Control and Prevention recommended that continuous variables be reported as mean±standard deviation and categorical variables be expressed as proportions in all statistical studies. The median was used to fill in missing values for continuous variables, while the distribution of the variables’ available instances was used to fill in missing values for categorical variables [25, 26]. The t-test (continuous variables) and the chi-square test (categorical variables) were employed to evaluate between-group differences between individuals categorized for hyperlipidemia. Three alternative models of blood Pb, Cd, and Se, were examined for their effects on hyperlipidemia using multivariate logistic regression. There is no covariate adjustment in the crude model. Gender, age, and race were modified in a minimally adjusted model. For a fully adjusted model, modifications were made for gender, age, race, education level, marital status, diabetes, hypertension, smoking status, and alcohol use status. Additionally, blood Pb, Cd, and Se levels were converted by the ln function in the regression analysis (ln transform) due to the skewed blood Pb, Cd, and Se levels. The model was used to determine the odds ratios (OR) and 95% confidence intervals (CI) for the relationship between blood Pb, Cd, and Se levels and hyperlipidemia. To test for heterogeneity of connections among subgroups, subgroup analyses of the relationship between blood Pb, Cd, and Se levels and hyperlipidemia were carried out with prespecified possible effect modifiers that included interaction effects. A threshold effects analysis model examined associations and inflection points between blood Pb, Cd, and Se levels and hyperlipidemia. R version 4.2.2 (http://www.R-project.org) and Empower software (www.empowerstats.com) were used for all analyses. ***P***<0.05 was used as the statistical significance level.

## Results

### Baseline participant characteristics

There were 5429 participants, with a mean age of 53.70±16.63 years and a gender split of 47.1% men and 52.9% women. Blood Pb, Cd, and Se concentrations were 1.41±1.52, 0.51±0.58, and 194.00±28.15, respectively, in terms of mean values. Of the subjects, hyperlipidemia affected 67.84% of them.

The clinical characteristics of the individuals are shown in Table 1 as a column variable stratified by hyperlipidemia. Age, gender, race, marital status, education level, smoking status, alcohol use status, body mass index, hypertension, diabetes, family Size, blood Pb, Cd, and Se were statistically significant in the presence or absence of hyperlipidemia (***P***<0.05). Hyperlipidemic patients had higher blood Pb and Se levels than non-hyperlipidemic patients. They were likelier to be older, male, non-Hispanic White, high school graduate/GED or equivalent, married/living with partner, and body mass index<30 kg/m^2^.

**Table 1:** Characteristics of the study population based on hyperlipidemia.

Variables	Non-hyperlipidemia group(N = 1746)	Hyperlipidemia group(N = 3683)	*P-*value
Age (years)	46.22 ± 17.26	57.25 ± 15.08	<0.001
Gender, %(SE)			0.014
Male	44.67%	48.25%	
Female	55.33%	51.75%	
Race, %(SE)			<0.001
Mexican American	10.37%	11.19%	
Other Hispanic	9.62%	11.11%	
Non-Hispanic White	36.31%	40.43%	
Non-Hispanic Black	27.21%	20.20%	
Other Races	16.49%	17.08%	
Marital status, %(SE)			<0.001
Married/Living with partner	59.16%	62.37%	
Widowed/Divorced/Separated	18.79%	26.05%	
Never married	22.05%	11.57%	
Education level, %(SE)			<0.001
Less than 9th grade	6.19%	10.16%	
9-11th grade	10.49%	11.33%	
High school graduate/GED or equivalent	19.44%	22.62%	
Some college or AA degree	32.86%	29.28%	
College graduate or above	31.02%	26.62%	
Smoking status, %(SE)			<0.001
Yes	39.52%	45.07%	
No	60.48%	54.93%	
Alcohol use status, %(SE)			0.003
Yes	13.24%	16.73%	
No	86.76%	83.27%	
Body mass index, %(SE)			<0.001
0 -25kg/m^2^ normal	34.31%	23.56%	
25-30kg/m^2^ overweight	30.18%	34.35%	
≥30kg/m^2^ obese	35.50%	42.09%	
Hypertension, %(SE)			<0.001
Yes	29.05%	50.88%	
No	70.95%	49.12%	
Diabetes, %(SE)			<0.001
Yes	8.64%	21.66%	
No	91.36%	78.34%	
Family income ($), %(SE)			0.181
< 15,000	15.44%	14.20%	
15,000–44,999	34.37%	37.42%	
45,000–99,999	29.29%	28.81%	
≥ 100,000	20.90%	19.57%	
Family size, %(SE)			<0.001
1	20.62%	22.37%	
2–4	57.79%	61.66%	
>4	21.59%	15.97%	
Trace element (SD)			
Blood lead (ug/dL)	1.22 ± 1.28	1.50 ± 1.61	<0.001
Blood cadmium (ug/L)	0.49 ± 0.59	0.52 ± 0.58	<0.001
Blood selenium (ug/L)	190.16 ± 25.39	195.82 ± 29.20	<0.001

Mean±SD for continuous variables: The weighted linear regression model calculated the ***P*** value.

(%) The ***P*** value was calculated using the weighted chi-square test for categorical variables.

### Association between hyperlipidemia and blood trace elements

The findings of the multiple logistic regression study on the correlation between blood Pb, Cd, and Se levels and hyperlipidemia are shown in Table 2. According to our findings, greater blood Pb, Cd, and Se levels are linked to a higher risk of developing hyperlipidemia. Blood Pb, Cd, and Se levels were all significantly correlated with an elevated risk of hyperlipidemia in our crude model (OR:1.64, 95% CI:1.50–1.79; OR:1.14, 95% CI:1.06–1.22; and OR:4.96, 95% CI:3.19–7.70). The connection between blood Se level and an elevated risk of hyperlipidemia was strong and significant when age, gender, and race were considered in a minimally adjusted model (Se: OR:6.38, 95% CI:3.94–10.31, ***P***<0.001). Blood Pb and Se levels and the risk of developing hyperlipidemia remained strong and significant after accounting for all covariates in a fully adjusted model (Pb: OR:1.50, 95% CI:1.28–1.76, ***P***<0.001; Se: OR:4.96, 95% CI:2.27–10.82, ***P***<0.001). Blood Cd, however, had no statistically significant effect in minimally adjusted and fully adjusted models. Using quartiles, we analyzed sensitivity and converted blood Pb, Cd, and Se levels from continuous to categorical variables. In the Crude model, we discovered that participants with blood Pb, Cd, and Se concentrations in the Q2(Pb: OR:1.72, 95% CI:1.47–2.01; Cd: OR:1.18, 95% CI:1.00–1.38; Se: OR:1.19, 95% CI:1.02–1.40), Q3(Pb: OR:2.13, 95% CI:1.82–2.51; Cd: OR:1.29, 95% CI:1.10–1.52; Se: OR:1.39, 95% CI:1.18–1.63), and Q4(Pb: OR:2.46, 95% CI:2.09–2.90; Cd: OR:1.36, 95% CI:1.15–1.60; Se: OR:1.80, 95% CI:1.53–2.12) groups had a higher and statistically significant risk of hyperlipidemia (All ***P*** for trend<0.001). Interestingly, in the minimally adjusted model, the probability of hyperlipidemia was significantly correlated with blood Pb and Cd concentrations in groups Q2, Q3, and Q4. However, this correlation was not statistically significant (All ***P*** > 0.05). However, blood Se levels in groups Q2(OR:1.24, 95% CI:1.05–1.46), Q3(OR:1.48, 95% CI:1.25–1.76), and Q4(OR:1.92, 95% CI:1.62–2.29) were significantly and consistently related to a reduced incidence of hyperlipidemia(***P*** for trend<0.01). In fully adjusted model, blood Pb and Se concentrations were significantly correlated with the possibility of hyperlipidemia in Q3(Pb: OR:1.65, 95% CI:1.24–2.19; Se: OR:1.46, 95% CI:1.10–1.92) and Q4(Pb: OR:2.12, 95% CI:1.56–2.88; Se: OR:1.84, 95% CI:1.38–2.45) groups, with statistically significant and consistent results(All ***P*** for trend 0.001); however, participants in the Q2 group had statistically significant 36% increase in the risk of blood Cd concentration and hyperlipidemia compared to participants in the Q1 group(OR:1.36, 95% CI:1.03–1.79, ***P*** = 0.03).

**Table 2:** Associations between blood metals (ln transform) and hyperlipidemia among US adults.

	Crude model		Minimally adjusted model		Fully adjusted model	
	OR (95% CI)	*P*-value	OR (95% CI)	*P*-value	OR (95% CI)	*P*-value
**Blood lead (ug/dL)**						
** Continuous**	1.64 (1.50, 1.79)	<0.001	1.08 (0.97, 1.19)	0.145	1.50 (1.28, 1.76)	<0.001
** Quartile**						
** Q1(-2.995 - -0.400)**	1		1		1	
** Q2(-0.385–0.058)**	1.72 (1.47,2.01)	<0.001	1.16 (0.98, 1.38)	0.082	1.27 (0.97, 1.67)	0.081
** Q3(0.067–0.494)**	2.13 (1.82,2.51)	<0.001	1.18 (0.99, 1.42)	0.066	1.65 (1.24, 2.19)	<0.001
** Q4(0.500–3.660)**	2.46 (2.09,2.90)	<0.001	1.15 (0.95, 1.39)	0.148	2.12 (1.56, 2.88)	<0.001
** OR for trend**	1.82 (1.64, 2.02)		1.09 (0.97, 1.24)		1.65 (1.36, 2.00)	
***P* for trend**	<0.001		0.152		<0.001	
**Blood cadmium (ug/L)**						
** Continuous**	1.14 (1.06, 1.22)	<0.001	1.01 (0.93, 1.08)	0.986	1.07 (0.93, 1.22)	0.337
** Quartile**						
** Q1(-2.659 - -1.660)**	1		1		1	
** Q2(-1.609 - -1.139)**	1.18 (1.01, 1.38)	0.047	0.93 (0.79, 1.11)	0.440	1.36 (1.03, 1.79)	0.030
** Q3(-1.108 - -0.562)**	1.29 (1.10, 1.52)	0.002	0.84 (0.70, 1.10)	0.051	1.24 (0.94, 1.65)	0.134
** Q4(-0.544–2.230)**	1.36 (1.15, 1.60)	<0.001	1.01 (0.84, 1.19)	0.980	1.25 (0.92, 1.70)	0.156
** OR for trend**	1.17 (1.08, 1.27)		0.99 (0.91, 1.08)		1.11 (0.95, 1.29)	
***P* for trend**	<0.001		0.888		0.204	
**Blood selenium (ug/L)**						
** Continuous**	4.96 (3.19, 7.70)	<0.001	6.38 (3.94, 10.31)	<0.001	4.96 (2.27, 10.82)	<0.001
** Quartile**						
** Q1(4.497–5.176)**	1		1		1	
** Q2(5.176–5.254)**	1.19 (1.02,1.40)	0.026	1.24 (1.05, 1.46)	0.013	1.21 (0.92, 1.59)	0.180
** Q3(5.254–5.335)**	1.39 (1.18, 1.63)	<0.001	1.48 (1.25, 1.76)	<0.001	1.46 (1.10, 1.92)	0.008
** Q4(5.335–6.540)**	1.80 (1.53, 2.12)	<0.001	1.92 (1.62, 2.29)	<0.001	1.84 (1.38, 2.45)	<0.001
** OR for trend**	8.04 (4.58, 14.10)		10.39 (5.69,18.99)		9.03 (3.37, 24.18)	
***P* for trend**	<0.001		<0.001		<0.001	

Minimally adjusted for age, gender, and race.

Fully adjusted for age, gender, race, marital status, education level, smoking status, alcohol use status, body mass index, hypertension, diabetes, family income, and family size.

### Analysis of subgroups

**Fig 2:**
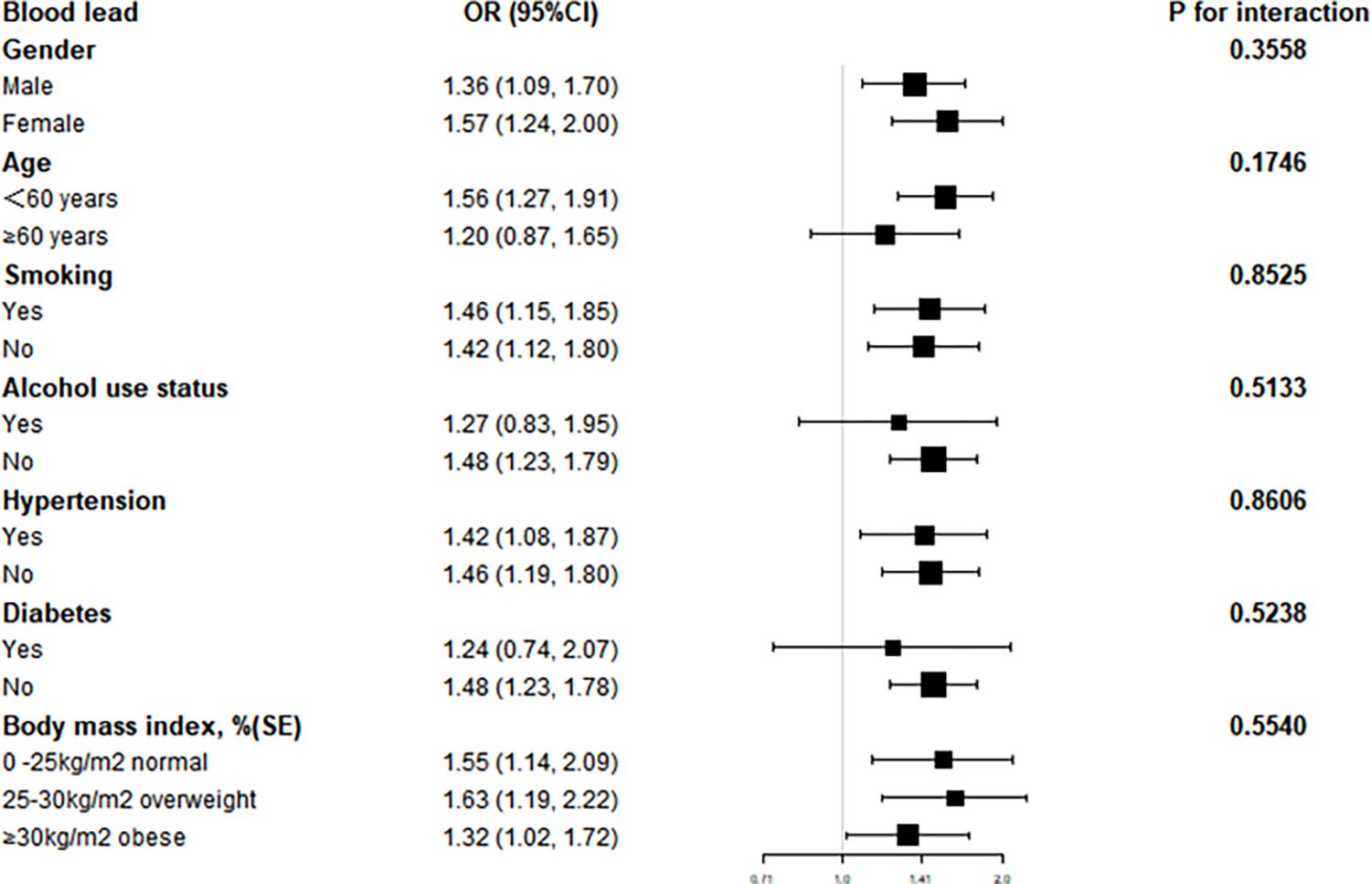
Subgroup analysis of blood lead.

**Fig 3:**
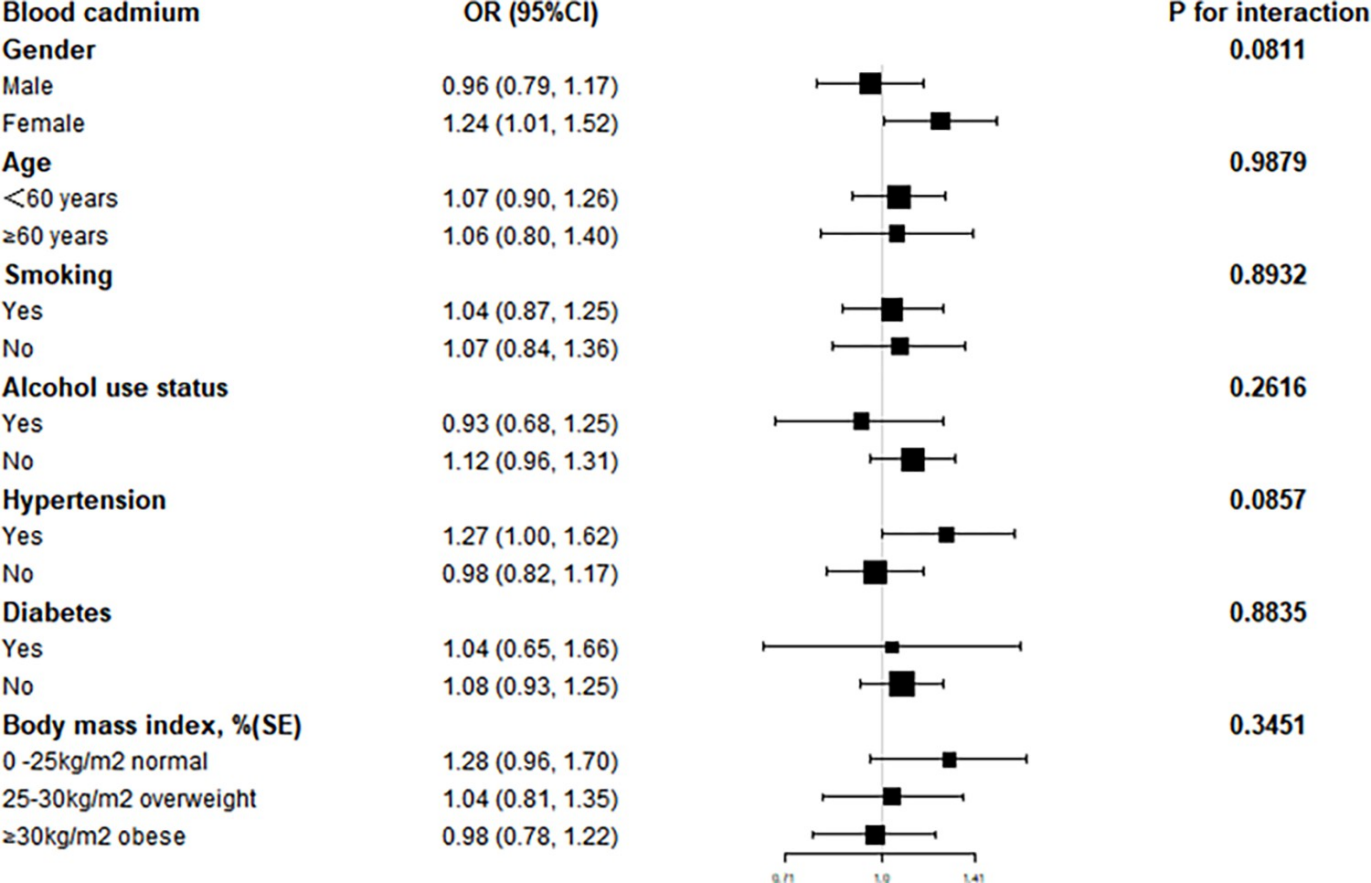
Subgroup analysis of blood cadmium.

**Fig 4:**
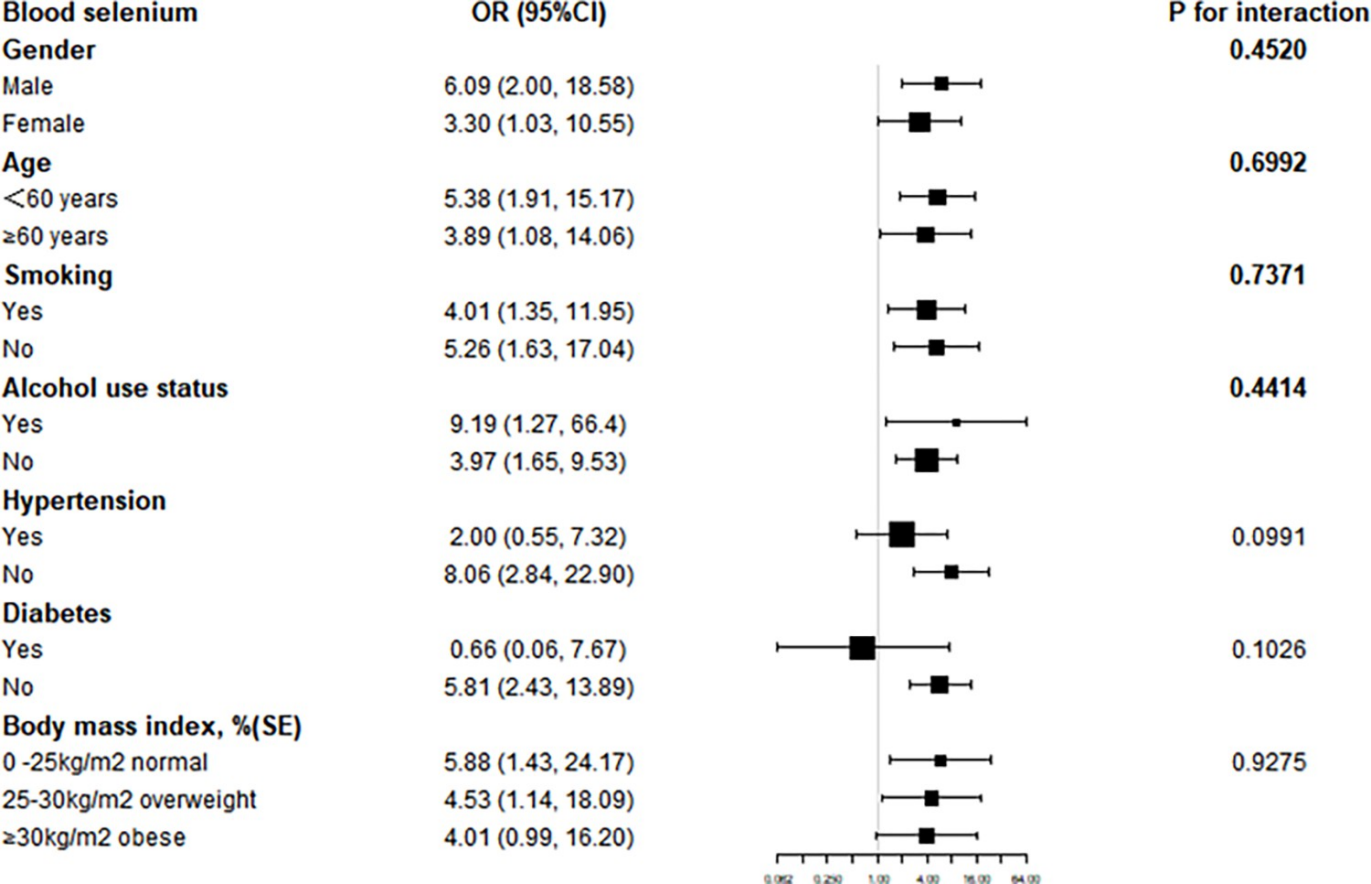
Subgroup analysis of blood selenium.

Subsequent subgroup analyses revealed that the link between blood metals and hyperlipidemia was erratic, as shown in Figs 2–4. They allowed us to investigate potential variables impacting the association between blood metals and the signs and symptoms of hyperlipidemia. In individuals with gender, smoking status, hypertension, body mass index, age<60, no alcohol, and no diabetes, we discovered a significant positive connection between blood Pb and the likelihood of hyperlipidemia(***P***<0.05) (Fig 2). Contrarily, there was a significant positive association between blood Cd level and the risk of hyperlipidemia in females, as well as no alcohol use status, hypertension, and 0<body mass index<25 kg/m^2^(***P***<0.05) (Fig 3). Pleasantly, there was a significant positive connection between blood Se level and the risk of hyperlipidemia in gender, age, smoking status, alcohol use status, body mass index, no hypertension, and no diabetes (***P***<0.05) (Fig 4). The results of an interaction test revealed that there was no statistically significant difference between categories in the relationship between blood metals and hyperlipidemia, implying that factors such as gender, age, smoking status, alcohol use status, hypertension, diabetes, and body mass index had no appreciable influence (***P*** for interaction > 0.05).

**Fig 5:**
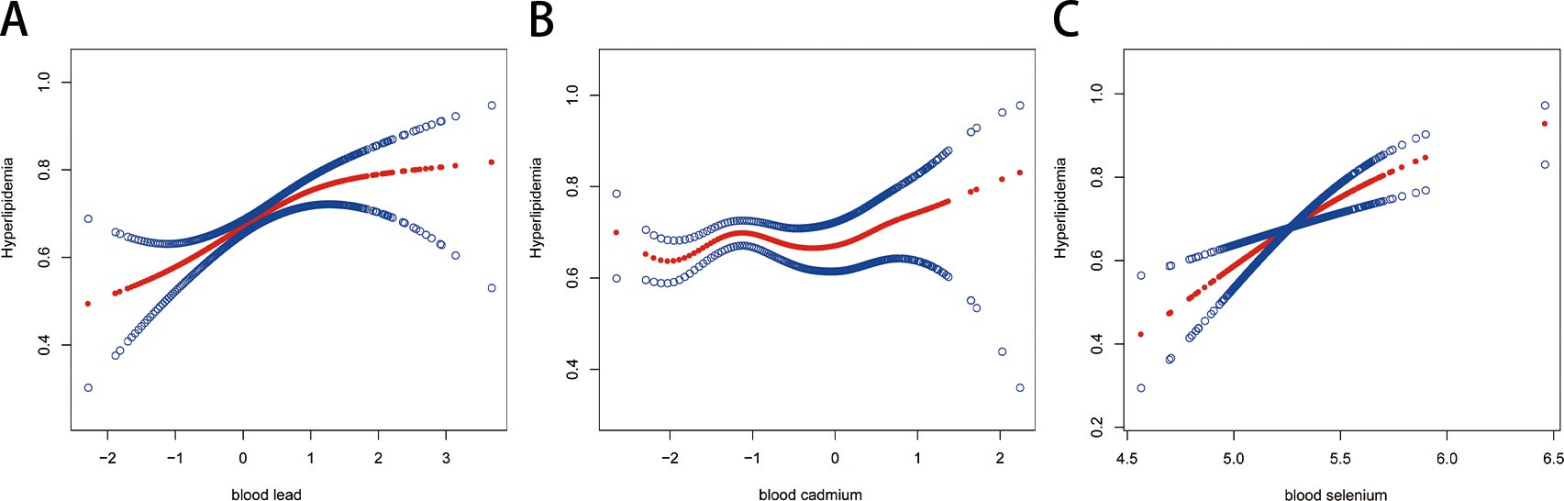
Smooth curve fitting.

The smoothed curve fitting method was then used to depict the nonlinear association between blood Pd, Cd, and Se levels and hyperlipidemia, as seen in Fig 5. Adjusting factors: age, gender, race, marital status, education level, smoking status, alcohol use status, body mass index, hypertension, diabetes, family income, and family size. We discovered a positive correlation between blood lead, cadmium, and selenium levels and the risk of hyperlipidemia using a two-stage linear regression model; however, this correlation was not statistically significant. It appeared to have a threshold effect, with fold points of 1.03, -2.21, and 5.14, respectively (log-likelihood ratio tests of 0.082, 0.149, and 0.118; Table 3).

**Table 3:** Threshold effect.

Outcome:	Hyperlipidemia risk		
	Blood lead	Blood cadmium	Blood selenium
Model I			
A straight-line effect	1.45 (1.22, 1.73)	1.08 (0.93, 1.24)	4.55 (2.03, 10.18)
Model II			
Fold points (K)	1.03	-2.21	5.14
< K-segment effect 1	1.58 (1.30, 1.92)	0.43 (0.12, 1.50)	0.59 (0.04, 8.78)
> K-segment effect 2	0.86(0.48, 1.55)	1.12 (0.96, 1.30)	7.68 (2.69, 21.96)
Effect size difference of 2 vs. 1	0.55 (0.28, 1.05)	2.63 (0.71, 9.72)	13.03 (0.52, 327.63)
Equation predicted values at breakpoints	1.37 (1.18 1.57)	0.64 (0.47, 0.82)	0.46 (0.29, 0.62)
Log likelihood ratio tests	0.079	0.141	0.115

Result variable: Hyperlipidemia

Exposure variables: Blood lead, Blood cadmium, Blood selenium

Results are expressed as OR (95%CI).

Adjusted for age, gender, race, Marital status, education level, Smoking status, Alcohol use status, Body mass index, Hypertension, and Diabetes.

## Discussion

Using data from the National Health Survey for 2011–2012, 2013–2014, 2015–2016, and 2017–2018, this study examines the connection between metals and hyperlipidemia in American adults. The levels of lead, cadmium, and selenium in the blood were independently associated with hyperlipidemia in this investigation. Our findings imply that the relationship between blood lead, cadmium, and selenium levels and hyperlipidemia becomes more evident after total adjustment. The interaction test and subgroup analysis results also demonstrated that this link was the same across populations.

To the greatest extent of what we know, research has discovered connections between blood lead, cadmium, and selenium levels and hyperlipidemia. Multiple metals have also been linked to lipid indicators under various exposure situations [27–30]. For instance, co-exposed populations to heavy metals were divided into high- and low-exposure groups using an unsupervised clustering method proposed by Yao et al., with the high-exposure group showing higher metal concentrations as well as significantly higher Gamma-glutamyl transferase (GGT), Systolic blood pressure (SBP), Diastolic blood pressure (DBP), and mortality rates [31]. In a cross-sectional investigation conducted in northeastern China, dyslipidemia biomarkers were linked to heavy metal exposure [32]. In research, blood lead exhibited a linear dose-response association with all-cause mortality and cardiovascular disease mortality with a median follow-up time of 136 months [33]. According to Wan et al., blood lead and metal mixtures of essential metals were positively correlated with all hyperlipidemia profiles in both the weighted quantile sum (WQS) regression model and the Bayesian kernel machine regression (BKMR) model, and blood lead and metal mixtures of essential metals were positively associated with blood total cholesterol, LDL cholesterol, and HDL cholesterol in a multivariate linear regression model (All ***P***<0.05) [34]. Increased blood levels of lead, mercury, and cadmium significantly increase the odds of having high total cholesterol in the US population after adjusting for age, sex, and socioeconomic status, according to the 2009–2012 National Health and Nutrition Examination Survey by Buhari et al. [35]. In the United States, adults’ serum total cholesterol, LDL cholesterol, HDL cholesterol, triacylglycerol, and Apo B and Apo A-I concentrations were all higher when their serum selenium levels were higher, according to research by Bleys et al. [36]. High blood selenium levels are linked to higher LDL and total cholesterol levels in American adults. Selenium was only linked to higher HDL cholesterol at low selenium levels [37]. Research of the entire population found a positive correlation between serum selenium levels and LDL, total, and total cholesterol [38]. According to Lin, higher levels of lead and cadmium are linked to dyslipidemia and elevated CIMT [39]. Messner et al. demonstrated that cadmium is a novel independent risk factor for early atherosclerotic processes and in vivo correlations, the same as our results [40]. According to the results of our study, elevated blood levels of lead, cadmium, and selenium were linked to a greater risk of hyperlipidemia. The blood lead level(OR:1.64, 95% CI:1.50–1.79), the blood cadmium level(OR:1.14, 95% CI:1.06–1.22), and the blood selenium level (OR:4.96, 95% CI: 3.19–7.70) were all significantly correlated with a greater likelihood of hyperlipidemia (All ***P***<0.001) in crude model; In the fully adjusted model, the link between blood lead and blood selenium levels and an elevated risk of hyperlipidemia was still solid and significant(Pb: OR:1.50, 95% CI:1.28–1.76, ***P***<0.001; Se: OR:4.96, 95% CI:2.27–10.82, ***P***<0.001).

The concentrations of blood lead, cadmium, and selenium may influence hyperlipidemia through several possible pathways. First, there is a favorable correlation between cholesterolemia and the level of 8-epi-PGF2alpha, one of the indicators of oxidative stress [41]. When metals in the blood bind to sulfhydryl protein groups, reactive oxygen species are released, which causes oxidative stress in the organism and depletes glutathione levels [42]. After that, oxidative stress triggers an inflammatory response that leads to the development of hyperlipidemia [43]. Second, by encouraging the release of lipids from the liver and adipose tissues, metals in the blood may cause dyslipidemia by causing reduced antioxidant enzymes such as superoxide dismutase and catalase activity, elevated hepatic and renal lipid peroxide levels, and serum markers of abnormal hepatic function [44]. The oxidation of cell membranes caused by metals in the blood, such as lead, cadmium, and selenium, can potentially cause hepatotoxic effects [44], which impact liver function and lessen lipid production in hepatocytes [45, 46]. Xia et al., in their experimental studies, also revealed that changes in the composition and abundance of the intestinal microbiota, which may result in dysbiosis, and the transcript concentrations of genes related to lipid metabolism (glucokinase, carbohydrate regulatory element-binding protein, and pyruvate kinase) were all found to be significantly elevated in the livers of lead-treated mice [47].

This study has several advantages. First, a large sample size, appropriate covariate adjustment, and sample data from a four-period cross-section are used to improve the the purpose of analysis representativeness, statistical power, and reliability. Second, sensitivity analysis was utilized to lower the possibility of false positives. As this population may always have hypercholesterolemia and the impact of pregnancy on metal, we excluded people with atherosclerotic cardiovascular disease and those who were pregnant from this research. We then used quartiles to partition the metal concentrations in the blood, which boosted intergroup variability and enhanced interpretability. This study does have some drawbacks, in any case. First of all, because this was a cross-sectional study and the data was gathered at a particular time, it is impossible to say whether the exposure happened before the outcome or whether the outcome led to the exposure. Moreover, the results of the research only enable us to draw conclusions; they do not establish a causal link between the exposure and the outcome. Consequently, further prospective cohort research is required to elucidate causation. Second, despite measuring serum levels of pollutants and making several adjustments during the analysis to account for various confounding factors, other confounding factors, such as a history of long-term use of medications like steroids, fail to account for heavy metal metabolites and biomarkers, which may still affect the outcomes. We won’t be able to incorporate these confounders in our analysis since they are not listed in the NHANES database. Furthermore, some individuals may have been exposed to metals for extended periods before the survey, which might have skewed the results. Consequently, our study cannot completely account for the adverse effects of long-term exposure to metals on the body. Additionally, in that study, we only considered the individual impact of the three metallic elements on hyperlipidemia, and we did not feel any combined effects. Finally, although hyperlipidemia in persons under 60 years may be attributed to dietary type or lifestyle choices, hyperlipidemia in the senior population is more likely owing to changes in bodily functioning with age. We did age stratification to increase the direct impact of blood lead, cadmium, and selenium levels on hyperlipidemia. No study has yet been able to adequately explain the various biological mechanisms behind the development of coronary vascular disease by metal levels and the inherent relationship between such effects as age. The interactions between metals and disease are complicated and vary. As a result, it might not be suitable to generalize our findings.

This research analyzes results from a comprehensive nationwide survey in the US. After correcting for confounders, our results indicate a substantial correlation between blood lead, cadmium, and selenium levels and hyperlipidemia. Further study is required because these findings do not prove a causal link.
